# Mitochondrial Calcium Uniporter protects hippocampal CA2 neurons from NMDA-induced excitotoxic injury

**DOI:** 10.1186/s10020-026-01528-y

**Published:** 2026-06-08

**Authors:** Aleksandra Skweres, Małgorzata Beręsewicz-Haller, Omar Basheer, Martyna Nalepa, Aleksandra Owczarek, Jakub Szymanowski, Maria Kawalec, Joanna Gruszczyńska-Biegała, Barbara Zabłocka, Michał Węgrzynowicz

**Affiliations:** 1 Laboratory of Molecular Basis of Neurodegeneration, Mossakowski Medical Research Institute, Polish Academy of Sciences, Pawińskiego 5, Warsaw, 02-106 Poland; 2 Doctoral School of Translational Medicine, Centre of Postgraduate Medical Education, Marymoncka 99/103, Warsaw, 01-813 Poland; 3 Molecular Biology Unit, Mossakowski Medical Research Institute, Polish Academy of Sciences, Pawińskiego 5, Warsaw, 02-106 Poland; 4 Laboratory of Advanced Microscopy Techniques, Mossakowski Medical Research Institute, Polish Academy of Sciences, Pawińskiego 5, Warsaw, 02-106 Poland

**Keywords:** MCU, Hippocampus, CA2, Excitotoxicity, Neuroprotection, Mitochondrial calcium, Organotypic cultures

## Abstract

**Background:**

The hippocampal region CA2, unlike neighboring CA1, is exceptionally resistant to excitotoxicity, although the mechanisms behind this phenotype are unknown. Given the importance of mitochondrial calcium buffering, we investigated whether Mitochondrial Calcium Uniporter (MCU), known to be enriched in CA2, contributes to this resistance.

**Methods:**

We employed immunostaining techniques in rodent brain tissue and organotypic slice cultures to visualize MCU across hippocampal regions under resting and excitotoxic conditions. Subsequently, we pharmacologically modulated MCU in an organotypic model of hippocampal excitotoxicity to assess its contribution to regional resistance.

**Results:**

We found a strong spatial correlation between resistance to NMDA excitotoxic injury and MCU expression. NMDA exposure resulted in MCU upregulation in CA2, and pharmacological inhibition of MCU sensitized CA2 neurons to excitotoxic damage in a dose-dependent manner, while having minimal effect on the already vulnerable CA1 neurons, which express low MCU levels.

**Conclusions:**

MCU is known to exacerbate NMDA-induced cell injury, although our data demonstrate a functional association between MCU activity and CA2 neuroprotection, suggesting that CA2 neurons may possess unique mitochondrial calcium handling capabilities. Our study provides novel insight into mechanisms supporting CA2 resistance to NMDA excitotoxic death and emphasizes context-dependent roles of MCU in neuronal injury or survival.

## Background

Due to its small size and difficulties in clearly delineating its borders in non-labelled hippocampus, the CA2 region has often been omitted from hippocampal studies. Despite limited knowledge, it has long been known that CA2 pyramidal neurons, unlike those in adjacent CA1 and CA3 regions, exhibit unusually high synaptic stability, manifested by their resistance to induction of long-term potentiation (LTP) and depression (LTD), particularly at their proximal dendrites (Chevaleyre and Siegelbaum 2010). For years, the molecular basis of this phenotype, similarly to the specific role of CA2 in brain function remained unclear. However, recent emerging interest in this region has revealed its molecular uniqueness (Farris et al. 2019) and its critical importance for social recognition memory, the ability to distinguish individuals of the same species (Hitti and Siegelbaum 2014; Radzicki et al. 2025).

Additionally, several CA2-specific cellular mechanisms have recently been demonstrated to underlie the limited plasticity of this region’s neurons. These include inhibition of the H-Ras/ERK/MAP2K signaling cascade by the CA2-enriched protein, regulator of G-protein signaling 14 (RGS-14) (Lee et al. 2010). Another specific feature is the structure of the perineuronal net, that, unlike in CA1 or CA3, surrounds both dendrites and spines of CA2 neurons (Carstens et al. 2016). In addition, CA2 neurons exhibit attenuation of postsynaptic cytosolic Ca^2+^ transients (Simons et al. 2009), a crucial mechanism in LTP/LTD formation. Given mitochondria’s role in neuronal Ca^2+^ buffering (White and Reynolds 1997), the last mechanism may also involve the efficiency of mitochondrial Ca^2+^ uptake via the Mitochondrial Calcium Uniporter (MCU) a protein recently found to be highly enriched in CA2 neurons and implicated in their distinct electrophysiological properties (Farris et al. 2019; Pannoni et al. 2023, 2025).

In addition to limited synaptic plasticity, CA2 pyramidal neurons are uniquely resistant to injury and death in various neurological disorders, particularly those involving excitotoxicity. Numerous studies report a consistent pattern of hippocampal damage: CA1 is severely affected, CA3 moderately, and CA2 minimally or not affected at all. This has been observed in post-mortem brains of patients with stroke (Bartsch et al. 2015), temporal lobe epilepsy (Steve et al. 2014) or traumatic brain injury (Maxwell et al. 2003) as well as in corresponding animal models (Yang et al. 2000; Beręsewicz-Haller et al. 2021). The cellular basis of CA2 resistance remains unclear. While it has been proposed that the mechanisms underlying synaptic stability, such as specific Ca^2+^ dynamics, may also contribute to the protection against excitotoxicity (Leranth and Ribak 1991; Dudek et al. 2016), due to shared dependence on postsynaptic N-methyl-D-aspartate (NMDA) receptor (NMDAR) activity, this hypothesis has not been experimentally confirmed. To investigate this, we used hippocampal organotypic rat slice cultures to examine the role of MCU in CA2 resistance to excitotoxic damage.

## Methods

### Animals

The studies were performed using C57Bl/6J mice and Wistar rats. Animals were housed in the Animal Facility of the Mossakowski Medical Research Institute, Polish Academy of Sciences (MMRI PAS).

For the experiments described in this study, direct approval from the Local Ethics Committee was not required, as all data were obtained from tissues collected from animals that had not been subjected to any in vivo experimental procedures. Under the Act of 15 January 2015 on the protection of animals used for scientific or educational purposes, euthanasia performed solely for the collection of tissues for purposes specified in Article 3 of the Act, including scientific research, is not considered an experimental procedure and therefore does not require ethical approval, provided that the euthanasia method used is listed in Annex IV of Directive 2010/63/EU and is carried out by appropriately authorized personnel.

In the present study, tissues were collected from animals euthanized either by transcardial perfusion (adult rats and mice) or decapitation (newborn rat pups), both of which are methods listed in Annex IV of Directive 2010/63/EU. All procedures were performed by experienced personnel holding certificates issued by the Polish Laboratory Animal Science Association (PolLASA), as well as institutional authorization permitting mouse and rat euthanasia and tissue collection for experimental purposes using methods compliant with Annex IV of Directive 2010/63/EU.

### Organotypic hippocampal slice cultures

#### Establishment and maintenance of the cultures

Cultures were prepared as described by Beręsewicz-Haller and colleagues (Beręsewicz-Haller et al. 2021)with minor modifications. Hippocampi were isolated from 7-day-old rats (typically 6–8 animals per culture) and cut into 400 μm slices using a tissue chopper (McIlwain Tissue Chopper). Slices with preserved structure (verified under Primo Vert light microscope (Zeiss) were placed on Millicell-CM membrane inserts (Millipore, Cat# PICM03050) and cultured at 36 °C, 5% CO_2_ in humidified air. The culture medium contained 50% Neurobasal A medium (Cat# 10888022), 25% heat inactivated horse serum (Cat# 26050088), 20% Hanks’ Balanced Salt Solution (Cat# 24020091), 1% B27 supplement (Cat# 17504001), 25 mM 4-(2-Hydroxyethyl)-1-piperazineethanesulfonic acid (Cat# 15630056), 0.5 mM glutamax (Cat# 35050061) (all Gibco), glucose (2.56 mg/ml; Sigma-Aldrich) and 1% Antibiotic Antimycotic Solution (Sigma-Aldrich, Cat# A5955). Serum was gradually withdrawn and B27 increased between day in vitro (DIV) 3 and DIV7, after which cultures were maintained in serum-free medium supplemented with 2% B27.

#### Treatment

On DIV8, prior to the experiment, slices were incubated with non-toxic, low dose (2 µM) PI (propidium iodide; Sigma-Aldrich, Cat# P4170) for 30 min. to label and exclude any damaged slices from further processing. In the remaining slices, excitotoxicity was induced by a 3 h exposure to NMDA (Sigma-Aldrich, Cat# M3262) at 25 µmol/l (selective injury) or 100 µmol/l (global injury control) or 45 min. to glutamate (Glu) (Warchem, Cat# 55431) at 10 mmol/l (selective injury). MCU inhibition was performed with MCU-i4 (Tocris, Cat# 7195) at 2, 10 and 50 µmol/l (based on (Di Marco et al. 2020) for 4 h, either alone or with 25 µM NMDA or for 1 h 45 min with 10 mM Glu. For the combined treatment, cultures were preincubated with MCU-i4 for 1 h, then co-incubated with NMDA for 3 h or Glu for 45 min. Vehicle-treated slices served as negative controls. After treatment, NMDA-, MCU-i4- or Glu- free medium was applied and slices were maintained for another 20 h. Following the treatment, slices were stained with high dose (6 µM) PI for 1 h (20 h after NMDA exposure) to more thoroughly label injured neurons and estimate neuronal damage under each condition.

### Immunohistochemistry

#### Immunostaining of fixed brain sections

Adult mouse and rat brain sections were prepared and stained as described by Nalepa et al. with slight modifications (Nalepa et al. 2025). Animals were perfused with 4% paraformaldehyde (Lach-Ner), and their brains, following cryoprotection, were sectioned at 30 μm, using a Hyrax M25 microtome (Zeiss) with an MTR freezing unit (Slee Medical). Free-floating sections underwent antigen retrieval in 10 mM sodium citrate (Sigma-Aldrich), pH 6.0, at 80 °C, blocking with 5% normal horse serum (Vector Laboratories) and quenching of endogenous peroxidases with 3% H_2_O_2_ (Chempur) in 20% methanol (Pol-Aura). Sections were then incubated with rabbit anti-MCU antibody (Cell Signaling Technology Cat# 14997, 1:1000), followed by biotinylated anti-rabbit secondary antibody (Vector Laboratories, 1:2000), ABC-HRP kit (Vector Laboratories), biotinylated tyramide (IRIS Biotech) and re-incubated with ABC-HRP. Signal was visualized using the HRP DAB Substrate Kit (Vector Laboratories). Sections were counterstained with cresyl violet (Acros Organics), mounted with DPX (Merck) and examined under an Axiovert.A1 microscope (Zeiss).

#### Staining of organotypic slices

Directly after the 20 h recovery period following NMDA withdrawal (or at the corresponding time point in control conditions), slices were fixed in methanol for 24 h at -20 °C, then cut from the inserts together with underlaying membrane and stained as free-floating sections. After blocking, slices were incubated for 1 h at 37 °C with either anti-MCU (1:500) or anti-RGS-14 (BioLegend Cat# 821801, 1:200). Next, sections were incubated with Alexa Fluor-conjugated secondary antibodies (647 nm anti-rabbit, or 488 nm anti-mouse; Invitrogen, 1:1000). Nuclei were stained with DAPI (10 min.) and slices were mounted with FluorSave Reagent (Millipore).

### Confocal microscopy analysis

Microscopy was performed at the Laboratory of Advanced Microscopy Techniques, MMRI PAS. PI labelling was analysed using scans of single optical sections obtained under a 5x objective lens at an excitation wavelength of 514 nm and emission wavelength of 550 nm, with a laser power of 11% and a pinhole diameter of 7.95 AU using a confocal microscope (Zeiss LSM 510 Confocal Microscope), before and after slice exposure to NMDA, Glu and/or MCU-i4 by an experimenter blinded to the treatment conditions. Fixed organotypic slices stained for MCU or RGS-14 were analysed with a confocal microscope (Zeiss LSM 780 Confocal Laser Scanning Microscope) using 20x objective lens (excitation wavelength 633 nm, emission wavelength 697 nm; excitation wavelength 488 nm, emission wavelength 538 nm, respectively). Images were processed with ZEN 3.5 Lite software. To assess protein (immunostaining) or damage (PI staining) distribution across CA1-CA3 subregions, fluorescence intensity was measured along a manually drawn, 15 pixel-thick line over the pyramidal layer, and plotted as signal intensity vs. distance. CA2 was identified based on established anatomical and morphological criteria, including the position relative to the dentate gyrus, greater width of the pyramidal cell layer compared to CA1, and larger neuronal cell body size. In selected experiments, CA2 identity was additionally confirmed by RGS-14 immunostaining.

For quantitative analysis of MCU immunofluorescence in the CA2 region, a mask for the CA2 hippocampal subfield was manually delineated from microscopic images. The CA2 region was expanded by 50 pixels using a Euclidean distance transform to include adjacent tissue. For both the original region of interest (ROI) and its expanded region, pixel intensity histograms were computed and modelled using Gaussian distributions. The distributions from the ROI and its expanded region were compared, and their overlap was used to estimate an intensity threshold separating foreground signal from background. This threshold was used to segment object-related signal while reducing contributions from background, imaging artefacts, and non-specific staining. Residual outlier intensities were removed using a robust median absolute deviation filter with a threshold of 3.5. The remaining pixel intensities were rescaled using min–max normalization to a common intensity range (0–255) to reduce inter-sample variability in signal amplitude. Following thresholding, outlier removal, and normalization, the remaining pixels were used to compute mean intensity values and ROI area for the CA2 subfield. All analyses were performed by an experimenter blinded to the treatment conditions.

For quantitative assessment of neuronal damage, mean PI signal intensities were calculated for CA1 and CA2 to compare damage between treatment groups.

### Statistics

Each culture was treated as a biological replicate, with values averaged from technical replicates (individual slices per condition per culture). Biological replicates were used for statistical analysis. Statistical analysis of obtained data was performed using GraphPad Prism 10 (GraphPad Prism). Two-way repeated-measure analysis of variance (ANOVA) was applied to identify effects of treatment, region or treatment x region interaction. Both factors – region and treatment were treated as within-subject variables, as measurements were obtained from both CA1 and CA2 under all concentrations within each of seven analysed cultures. Tukey’s post-hoc test was used for individual comparisons between experimental groups. A linear regression analysis was performed to evaluate the dose-response relationship between inhibitor concentrations and mean cell damage. The goodness-of-fit was assessed using the coefficient of determination (R²) and the p-value for the slope. Data are presented as means of biological replicates ± SEM. *p* < 0.05 was considered statistically significant.

## Results and discussion

**Fig. 1:**
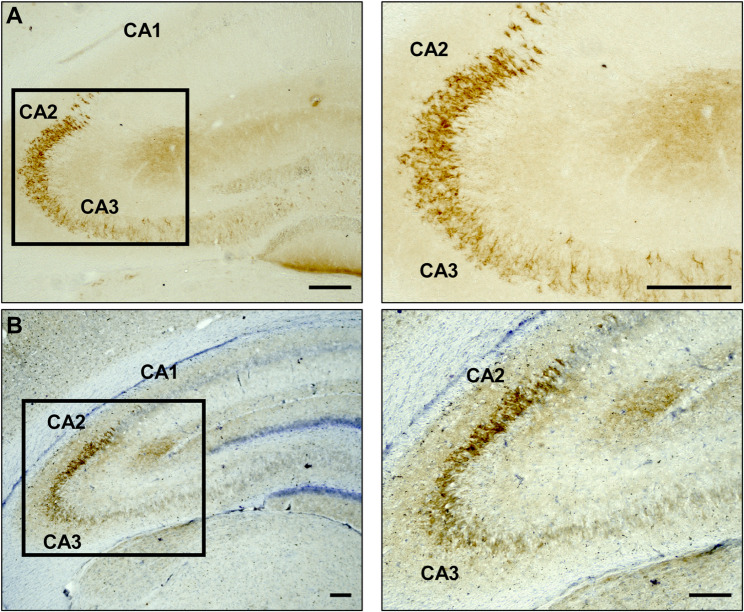
MCU protein distribution in mouse and rat hippocampus.** (A)** MCU immunostaining was performed on coronal sections of mouse hippocampus and visualized using diaminobenzidine (DAB, brown). Left: representative image showing CA1, CA2 and CA3 regions. Right: magnified view of the MCU-enriched area marked in the left panel. Scale bars: 200 μm. **(B)** MCU immunostaining was performed on coronal sections of rat hippocampus and visualized using DAB (brown). Nuclei were counterstained with cresyl violet (purple). Left: representative image showing CA1, CA2 and CA3 regions. Right: magnified view of the MCU-enriched area marked in the left panel. Scale bars: 200 μm

Recent studies report heterogeneous MCU mRNA (Farris et al. 2019) and protein distribution (Pannoni et al. 2023) across CA subregions in mice, with high expression in CA2, moderate in CA3 and low in CA1. We confirm this pattern in both mouse (Fig. 1A) and rat (Fig. 1B) brains, showing that MCU distribution correlates with pyramidal neurons resistance to pathology (see Background), suggesting a neuroprotective role of MCU.

### Organotypic hippocampal slices preserve in vivo vulnerability patterns and regional MCU distribution

**Fig. 2:**
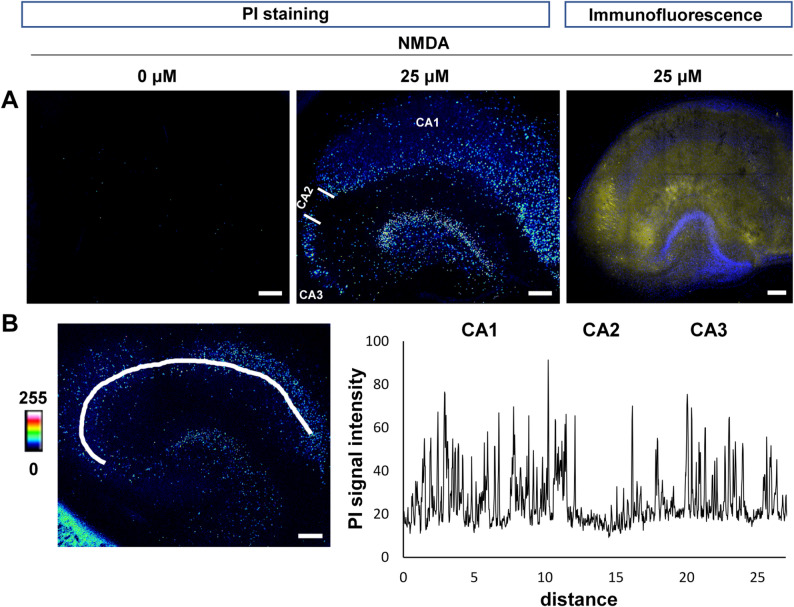
Differential sensitivity of hippocampal regions to excitotoxic stimuli in organotypic cultures.** (A)** Organotypic rat hippocampal slices were exposed to either vehicle (left) or 25 µM NMDA (middle and right) and stained with PI (pseudocolored for intensity ((low) 0-255 (high)) to visualize injured cells (left and middle). Following PI imaging, NMDA-treated slices were fixed and immunostained for the CA2 neuronal marker, RGS-14 (yellow) and stained with nuclei marker, DAPI (blue) (right). Representative images are shown. Scale bars: 200 μm. **(B)** Fluorescence intensity of PI staining was measured along a manually drawn line over the pyramidal layer in NMDA-treated slices (left; pseudocolored for intensity) and plotted as a function of distance (right). Representative image and corresponding graph are shown. Scale bar: 200 μm

For functional studies, we used a rat organotypic hippocampal slice culture model of excitotoxicity. Unlike dissociated cultures, organotypic slices preserve, to a large degree, hippocampal architecture and cell interactions (Stoppini et al. 1991). We first validated that our model retains region-specific sensitivity to excitotoxicity. Exposure to 25 µM NMDA caused a strong increase in PI signal in pyramidal layer of CA1, indicating extensive neuronal damage. In contrast, CA2 neurons in the same slices remained largely unaffected. Further staining of the slices for RGS-14, a CA2 marker (Lee et al. 2010), confirmed the identity of the resistant region (Fig. 2A). To precisely visualize the distribution of injured neurons, we quantified PI fluorescence along pyramidal layer. PI intensity was high in CA1 and moderately high in CA3, while CA2 showed only baseline signal with no major peaks indicating severely damaged neurons (Fig. 2B). These findings demonstrate that our model reproduces the spatial pattern of excitotoxic damage seen in human post-mortem tissue and in vivo disease models.

**Fig. 3:**
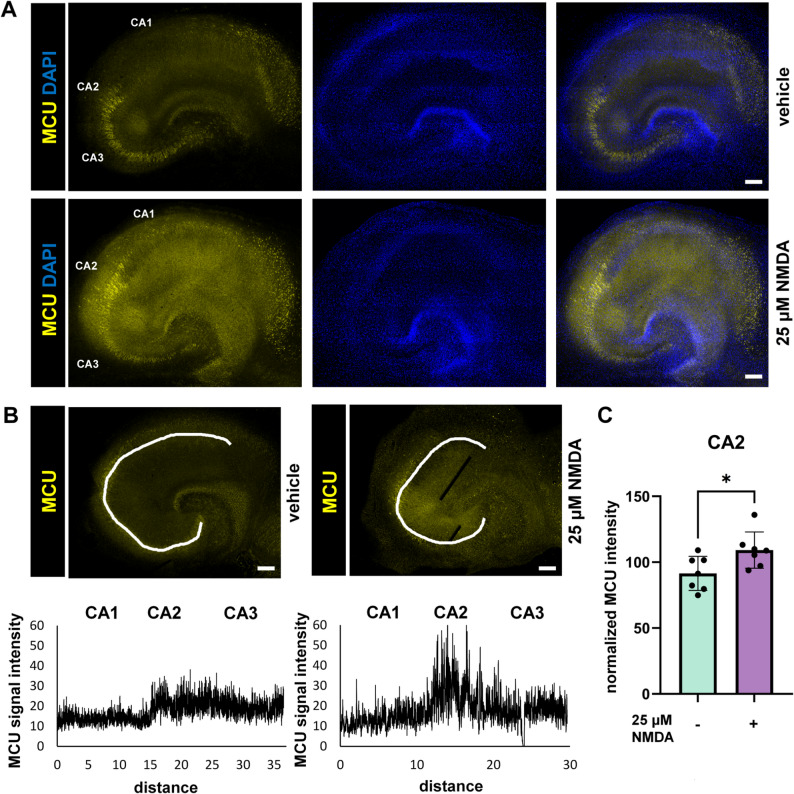
Changes in hippocampal MCU distribution in response to NMDA-excitotoxic stimuli in organotypic cultures. **(A)** Organotypic rat hippocampal slices were exposed to either vehicle (top) or 25 µM NMDA (bottom) and, following fixation, immunostained for MCU (yellow, left), labelled with the nuclei marker, DAPI (blue, middle) and merged (right). Representative images are shown. Scale bars: 200 μm. **(B)** Intensity of MCU immunofluorescent labelling was measured along a manually drawn line over the pyramidal layer in vehicle-treated (left) and NMDA-treated (right) slices and plotted as a function of distance (bottom). Representative images and corresponding graphs are shown. Scale bars: 200 μm. **(C)** Quantitative analysis of normalized MCU immunofluorescence intensity was performed for the CA2 region in the slices exposed to vehicle or 25 µM NMDA. Unpaired t-test revealed a significant increase in MCU protein expression in the CA2 region following 25 µM NMDA exposure (*p* = 0.029). Data are presented as means ± SEM. *n* = 7 biological replicates (individual cultures). *p < 0.05

To assess whether MCU distribution in organotypic slices reflects its native pattern in adult rodent brains, we examined MCU localization in vehicle-treated slices. We found that its distribution along the pyramidal layer closely mirrored the in vivo pattern: CA1 neurons showed minimal MCU immunoreactivity, while CA2 expressed very high levels of this protein. CA3 neurons showed a slightly lower MCU signal, with intensity gradually decreasing with the distance from the CA2/CA3 border (Fig. 3A and B). These data show that, in addition to the spatial pattern of excitotoxic damage, MCU distribution is also preserved in organotypic cultures. Moreover, the strong correlation between regional MCU content and resistance to NMDA-induced injury supports a potential role for MCU in mediating region-specific neuroprotection.

### NMDA induces selective MCU upregulation in CA2 neurons

In slices exposed to 25 µM NMDA, we observed a marked upregulation of MCU specifically in CA2 neurons (Fig. 3A and B), and quantitative analysis of the immunofluorescence signal confirmed a statistically significant ~ 20% increase in CA2 MCU protein expression under these conditions (Fig. 3C). This increase was detected at the experimental endpoint, i.e. after 3 h exposure to NMDA followed by 20 h of recovery, when neuronal injury was assessed by PI staining. However, since the slices were not analysed at earlier time points, the precise temporal alterations in MCU expression in this experimental setup remain unknown, and we cannot determine whether it represents an early or delayed response to NMDA stimulation. The CA2-specific NMDA-induced upregulation of MCU suggests a specific stress-related response in this neuronal population. However, based on the present data, we cannot determine whether this increase reflects transcriptional upregulation, altered MCU protein stability, increased mitochondrial content, or redistribution of mitochondria within CA2 neurons. Moreover, the observed increase may represent either an adaptive response contributing to CA2 resistance or a reactive change associated with NMDA-induced stress. Therefore, our data do not allow us to unequivocally conclude that MCU upregulation itself is causally responsible for the resistance of CA2 neurons.

Notably, this contrasts with previous findings in other neuronal populations, where MCU was shown to be downregulated in response to NMDA overstimulation as part of a protective feedback mechanism. For example, prolonged synaptic activity suppresses MCU expression in excitotoxicity-prone cortical and hippocampal primary neurons thereby limiting mitochondrial Ca^2+^ overload and reducing neuronal damage (Qiu et al. 2013). The CA2-specific NMDA-induced increase in MCU expression suggests a distinct response in neurons in this region, where MCU protein contributes to neuroprotection.

### MCU inhibition selectively sensitizes CA2 neurons to NMDA-induced injury

**Fig. 4:**
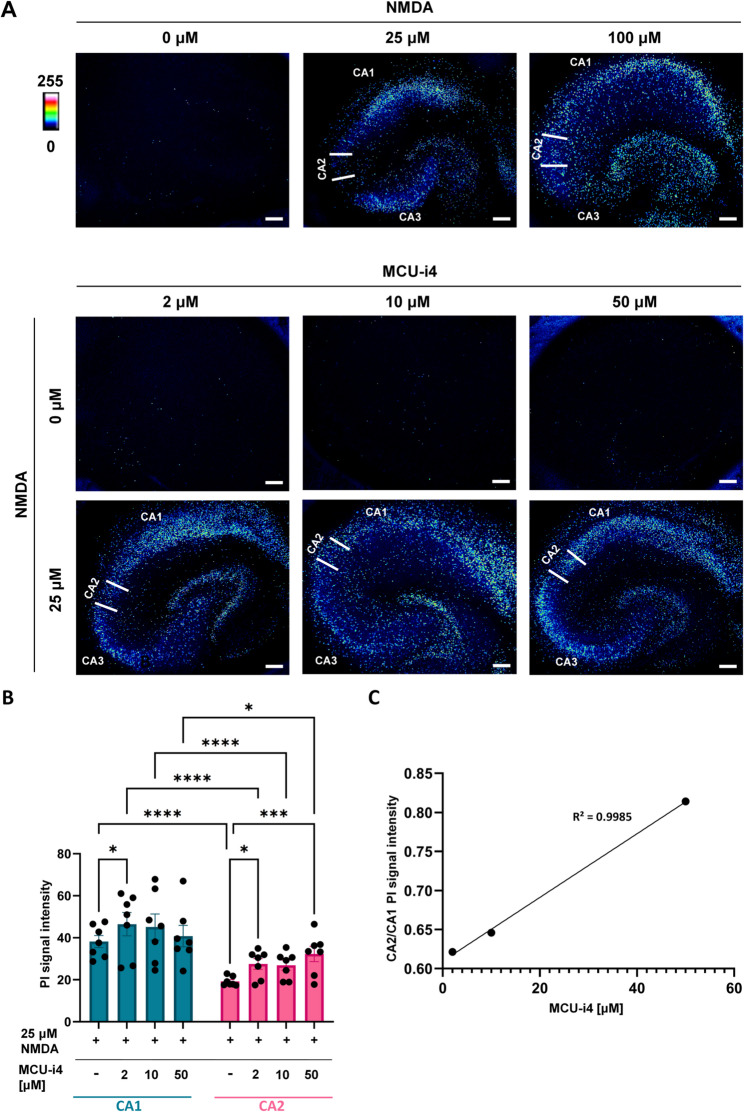
Effect of MCU inhibition on NMDA-induced damage in CA1 and CA2 regions in organotypic cultures.** (A) **Slices were exposed to vehicle (top left), NMDA (25 µM or 100 µM; top middle and right), MCU-i4 (2 µM; 10 µM or 50 µM; middle), or 25 µM NMDA combined with MCU-i4 (2 µM, 10 µM or 50 µM; bottom), and stained with PI (pseudocolored for intensity ((low) 0-255 (high)). Representative images are shown. Scale bars: 200 μm. **(B)** Average PI fluorescence intensity was quantified for CA1 and CA2 in slices exposed to 25 µM NMDA alone or in combination with MCU-i4 (2 µM, 10 µM or 50 µM). Two-way repeated-measure ANOVA revealed a main effect of treatment (F(3,18) = 3.761; *p* = 0.029), region (F(1,6) = 40.69; *p* = 0.0007) and a treatment x region interaction (F(3,18) = 4.65; *p* = 0.0141). Tukey’s test identified differences between CA1-25 µM NMDA vs. CA1-25 µM NMDA + 2 µM MCU-i4 (*p* = 0.0432), CA1-25 µM NMDA vs. CA2-25 µM NMDA (*p* < 0.0001), CA1-25 µM NMDA + 2 µM MCU-i4 vs. CA2-25 µM NMDA + 2 µM MCU-i4 (*p* < 0.0001), CA1-25 µM NMDA + 10 µM MCU-i4 vs. CA2-25 µM NMDA + 10 µM MCU-i4 (*p* < 0.001), CA1-25 µM NMDA + 50 µM MCU-i4 vs. CA2-25 µM NMDA + 50 µM MCU-i4 (*p* = 0.0371), CA2-25 µM NMDA vs. CA2-25 µM NMDA + 2 µM MCU-i4 (*p* = 0.0436) and CA2-25 µM NMDA vs. CA2-25 µM NMDA + 50 µM MCU-i4 (*p* = 0.0007). **(C)** Dose-response relationship was analyzed using the CA2/CA1 PI fluorescence intensity ratio to evaluate CA2 susceptibility relative to CA1 under increasing MCU-i4 concentrations combined with 25 µM NMDA. Mean CA2/CA1 ratios were 0.62 ± 0.04, 0.65 ± 0.05, and 0.81 ± 0.06 for 25 µM NMDA + MCU-i4 at 2 µM, 10 µM, and 50 µM concentrations, respectively. Linear regression revealed a positive correlation (R² = 0.9985, *p* = 0.025), indicating a linear dose-response relationship within the tested range. Data are presented as means ± SEM. *n* = 7 biological replicates (individual cultures). *p < 0.05, ***p < 0.001, ****p < 0.0001

To test the hypothesis that MCU supports CA2 neuron survival, we analysed NMDA-induced injury in CA regions following inhibition of MCU using MCU-i4. This compound binds to the Mitochondrial Calcium Uptake 1 protein – an endogenous inhibitor of MCU that activates the channel in response to elevated extramitochondrial Ca^2+^. By preventing its dissociation, the compound blocks MCU permeability even under high Ca^2+^ concentrations(Di Marco et al. 2020). First, we confirmed that MCU-i4 alone (2, 10 or 50 µM) caused no neuronal damage in CA1, CA2 or CA3 (Fig. 4A). However, when combined with 25 µM NMDA (a concentration non-damaging to CA2), MCU-i4 sensitized CA2 in a dose-dependent manner (Fig. 4A). Importantly, with the exception of the lowest, 2 µM MCU-i4 dose, the inhibitor had no effect on CA1 damage induced by 25 µM NMDA, aligning with the low MCU expression in that region and supporting its selective action in MCU-enriched CA2 (Fig. 4A). Two-way repeated-measure ANOVA of PI intensity apart from treatment effect identified also an effect of region and treatment x region interaction, proving the distinct response of CA1 and CA2 neurons to MCU-i4 + NMDA exposure (Fig. 4B). Post hoc direct comparisons showed that PI intensity was consistently higher in CA1 than CA2 in slices exposed to 25 µM NMDA alone or with 2, 10 or 50 µM MCU-i4. Despite this, MCU-i4 significantly increased PI signal in CA2 at 2 and 50 µM concentrations compared to NMDA alone (Fig. 4B). Notably, 2 µM MCU-i4 enhanced NMDA-induced damage in CA1, but this effect was absent at higher MCU-i4 concentrations (Fig. 4B). Since this effect was not reproduced at higher MCU-i4 concentrations and was not observed in the further Glu-induced excitotoxicity experiments, we attribute it to biological variability. However, as with any pharmacological approach, we cannot completely exclude potential off-target actions of MCU-i4 or indirect network-mediated effects that might contribute to the isolated increase in CA1 damage observed at the 2 µM concentration.

To better control for culture-to-culture and imaging-to-imaging variability, we analysed the CA2/CA1 PI signal ratio as an internal reference across MCU-i4 doses combined with 25 µM NMDA. Linear regression analysis revealed a significant positive correlation between inhibitor dose and the CA2-to-CA1 neuronal damage ratio (R² = 0.9985). This finding indicates a strong linear dose-response relationship within the tested range and confirming a differential MCU-i4-mediated sensitization effect in CA2 compared to CA1 (Fig. 4C).

### Association of MCU activity with CA2 resistance to NMDA-induced excitotoxicity

Our data appear to contrast with the common view that MCU activity via excessive entry of Ca^2+^ to mitochondria during excitotoxic events promotes neuronal injury and death. Prior studies with the use of different neuronal populations (Budd and Nicholls 1996; Stout et al. 1998) including hippocampal neurons (Qiu et al. 2013) showed that MCU knockout or inhibition reduced the excitotoxic damage, while its overexpression exacerbated cell injury. Our data indicate that this paradigm does not apply universally, and in specific cell types, MCU may serve a protective function. We show that this applies specifically to the pyramidal neurons of CA2 region, and we do not observe this effect in CA1 neurons, a population highly sensitive to excitotoxic insult. Together, while not yet defining the precise mitochondrial mechanisms involved, our findings, based on pharmacological MCU inhibition, suggest a strong association between exceptionally high MCU expression in CA2 neurons and the selective resistance of these cells to NMDA-mediated excitotoxic injury. The discrepancy between our findings and earlier work may result from limitations of dissociated hippocampal cultures. In such model, CA2 neurons possibly lose their regional identity or, even if they preserve it, it is masked by overwhelming majority of neurons from other populations. Meantime, organotypic cultures preserve hippocampal subfield architecture, enabling detection of unique, region-specific neuroprotective mechanisms such as that of MCU in CA2.

### Potential mechanisms of MCU-dependent neuroprotection in CA2

While the presented data are based on pharmacological MCU inhibition, without genetic manipulation of MCU expression or direct measurements of mitochondrial Ca²⁺ dynamics, it may still be hypothesized that CA2 pyramidal neurons possess unique adaptations that enable them to tolerate high mitochondrial Ca²⁺ loads without triggering cellular dysfunction. Notably, CA2 neurons contain more mitochondria than CA1 neurons, both in the soma (Farris et al. 2019) and dendrites, where they are also larger in size than those in CA1 dendrites (Pannoni et al. 2023). This suggests greater mitochondrial Ca^2+^ buffering capacity. Additionally, compared to CA1, CA2 neurons exhibit enhanced cytosolic Ca²⁺ handling. This includes more efficient Ca^2+^ extrusion (Simons et al. 2009) improved buffering, and significantly higher expression of Ca²⁺-binding proteins such as N-Terminal EF-Hand Calcium Binding Protein 1 (Sugita et al. 2002), calretinin (Seress et al. 1993), as well as parvalbumin and calbindin (Sloviter et al. 1991). These features may act jointly to reduce cytosolic Ca^2+^, even during prolonged cellular Ca^2+^ influx, such as during NMDAR overstimulation. As a result, extramitochondrial Ca^2+^ concentrations may remain lower in CA2 than in CA1, thereby protecting CA2 mitochondria from overload.

Why does pharmacological inhibition of MCU kill CA2 neurons, while protecting other neuronal populations? This may be due to cytosolic Ca^2+^ overload. Given efficient Ca²⁺ extrusion, buffering and binding, CA2 neurons could be especially sensitive to free cytosolic Ca²⁺ accumulation. Cytosolic Ca^2+^ alone, independent of mitochondrial signalling, can trigger neurotoxic cascades via activation of Ca^2+^-dependent enzymes such as endonucleases or calpains (Orrenius et al. 1989; Gerencser et al. 2009) Thus, the differing responses of CA1 and CA2 neurons to NMDA-mediated excitotoxicity and MCU inhibition may stem from varying sensitivities to cytosolic versus mitochondrial Ca^2+^ accumulation. Additionally, elevated MCU expression has been linked to enhanced mitochondrial bioenergetics, including more efficient oxidative phosphorylation and ATP production under conditions of high energetic demand (Cawley et al. 2026). Therefore, MCU inhibition in CA2 neurons may not only impair mitochondrial Ca^2+^ buffering, but also compromise mitochondrial energetic function during prolonged NMDAR overstimulation. As a consequence, this may increase neuronal vulnerability to cytosolic Ca^2+^-dependent neurotoxic processes. Still, these hypotheses require direct experimental verification In particular future studies should compare mitochondrial parameters between CA2 and CA1 neurons under NMDA excitotoxicity, including mitochondrial Ca^2+^ dynamics, mitochondrial membrane potential, electron transport chain activity, and ATP production. Such studies will help better define the role of MCU-mediated mitochondrial Ca^2+^ uptake in the differential vulnerability of these neuronal populations to NMDA-induced injury.

Although limited, some studies report neuroprotective roles of MCU. For instance, MCU protected phenochromocytoma PC12 cells from 1-methyl-4-phenylpyridinium-induced death (Wang et al. 2018). Also, glucocerebrosidase 1 knockout led to reduced MCU expression and greater Glu-induced cytosolic Ca^2+^ accumulation, exacerbating cell damage (Plotegher et al. 2020). Notably, both models relate to Parkinson’s disease, suggesting a potential role of MCU loss in its pathogenesis. These findings along with ours, indicate that the role of MCU in neuronal damage is not as unambiguous as it is commonly believed, and varies by cell type and condition.

### MCU inhibition does not sensitize CA2 neurons to Glu-induced injury

**Fig. 5:**
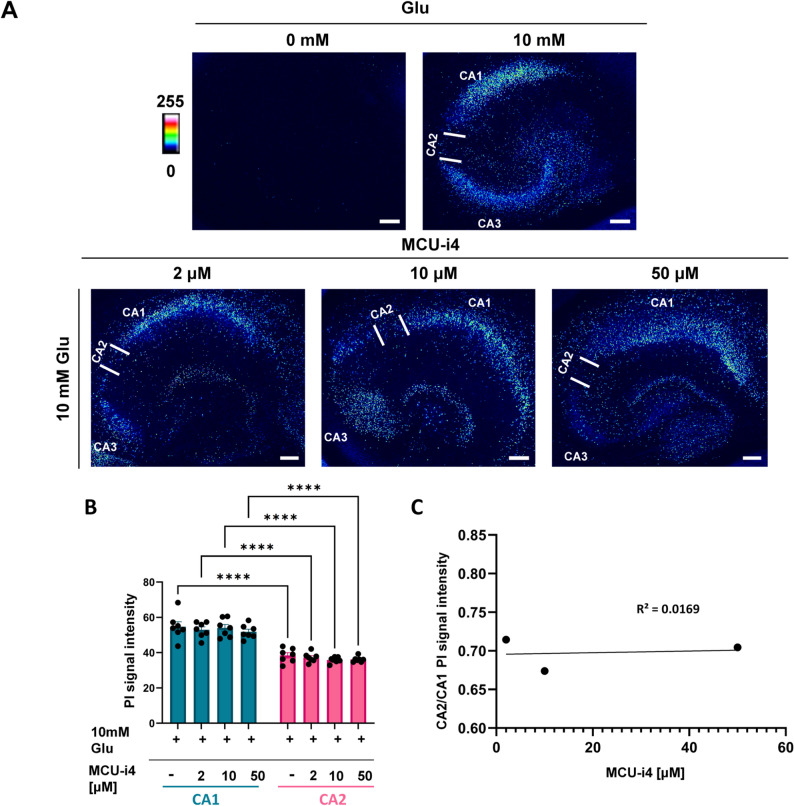
Lack of effect of MCU inhibition on Glu-induced damage in CA1 and CA2 regions in organotypic cultures.** (A)** Slices were exposed to vehicle (top left), 10 mM Glu (top right), or 10 mM Glu combined with MCU-i4 (2 µM, 10 µM or 50 µM; bottom), and stained with PI (pseudocolored for intensity ((low) 0-255 (high)). Representative images are shown. Scale bars: 200 μm. **(B)** Average PI fluorescence intensity was quantified for CA1 and CA2 in slices exposed to 10 mM Glu alone or in combination with MCU-i4 (2 µM, 10 µM or 50 µM). Two-way repeated-measure ANOVA revealed no effect of treatment (F(3,18) = 1.390; *p* = 0.2780), a main effect of region (F(1,6) = 129.0; *p* < 0.0001) and no treatment x region interaction (F(3,18) = 1.851; *p* = 0.1742). Tukey’s test identified differences between CA1-10 mM Glu vs. CA2-10 mM Glu (*p* < 0.0001), CA1-10 mM Glu + 2 µM MCU-i4 vs. CA2-10 mM Glu + 2 µM MCU-i4 (*p* < 0.0001), CA1-10 mM Glu + 10 µM MCU-i4 vs. CA2-10 mM Glu + 10 µM MCU-i4 (*p* < 0.0001) and CA1-10 mM Glu + 50 µM MCU-i4 vs. CA2-10 mM Glu + 50 µM MCU-i4 (*p* < 0.0001). **(C)** Dose-response relationship was analyzed using the CA2/CA1 PI fluorescence intensity ratio to evaluate CA2 susceptibility relative to CA1 under increasing MCU-i4 concentrations combined with 10 mM Glu. Mean CA2/CA1 ratios were 0.71 ± 0.07, 0.67 ± 0.05 and 0.70 ± 0.04 for 10 mM Glu + MCU-i4 at 2 µM, 10 µM, and 50 µM concentrations, respectively. Linear regression revealed no correlation between dose and response (R² = 0.0169 *p* = 0.9171). Data are presented as means ± SEM. *n* = 7 biological replicates (individual cultures). ****p < 0.0001

Next, to extrapolate the obtained data to more general excitotoxicity mechanisms, we assessed the effect of MCU inhibition on Glu-induced excitotoxicity. Organotypic hippocampal slices were exposed to 10 mM Glu for 45 min. and, similarly to the NMDA experiments, allowed to recover for 20 h following Glu withdrawal. We observed that this experimental paradigm, similarly to NMDA treatment, resulted in strong selective injury within the CA1 pyramidal layer, with weaker effects in CA3 and virtually no damage in CA2 (Fig. 5A). In contrast to NMDA, however, co-treatment with MCU-i4 at any of the applied concentrations (2 µM, 10 µM, or 50 µM) did not appear to induce detectable injury of CA2 pyramidal neurons (Fig. 5A). Quantification of PI signal intensity confirmed this observation, and two-way repeated-measures ANOVA revealed no significant effect of treatment, a significant main effect of region, and no treatment x region interaction. Direct post hoc comparisons showed no differences in PI signal intensity within either CA1 or CA2 between exposure groups, although PI signal remained consistently higher in CA1 than CA2 neurons under all treatment conditions (Fig. 5B), indicating that MCU inhibition did not modify the sensitivity of either CA1 or CA2 to 10 mM Glu. Consistently, linear regression analysis revealed a low correlation coefficient (R² = 0.1687) between MCU-i4 concentrations and the CA2/CA1 neuronal injury ratio (Fig. 5C). Taken together, these data clearly contrast with the earlier NMDA experiments, in which MCU inhibition sensitized CA2 neurons to excitotoxic injury. Instead, they indicate that under Glu exposure, MCU-i4 does not affect the characteristic CA1 vs. CA2 vulnerability pattern.

These data indicate that the involvement of MCU in CA2 neuronal resistance to excitotoxicity is preferentially associated with NMDAR-mediated injury rather than representing a universal mechanism engaged by all glutamatergic receptor types. Apart from NMDARs, Glu overexposure induces neuronal damage also through other receptor populations, including α-amino-3-hydroxy-5-methyl-4-isoxazolepropionic acid (AMPA) receptors, kainate receptors, and metabotropic glutamatergic receptors, and activation of different receptor classes may induce distinct patterns of hippocampal injury (Kristensen et al. 2001). In addition, although, to our knowledge, Ca^2+^ flux patterns have not been directly compared between different glutamatergic receptor-mediated excitotoxic paradigms in CA2 neurons, such differences have been reported in other experimental models. For example, stimulation of AMPA receptors in *Xenopus* spinal neurons produced approximately two-fold lower cytosolic Ca^2+^ elevations than NMDAR activation (Gleason and Spitzer 1998). Similarly, in mouse spinal neurons, NMDAR-dependent Ca^2+^ entry significantly exceeded that mediated by non-NMDAR receptors (Tymianski et al. 1993). Therefore, one may hypothesize that mixed activation of multiple glutamatergic receptor populations during Glu exposure generates Ca^2+^ responses with different magnitude, kinetics, and subcellular distribution compared with selective NMDAR overstimulation, potentially reducing the relative dependence on MCU-mediated mitochondrial Ca^2+^ buffering. Moreover, mitochondrial Ca^2+^ buffering has been shown to depend strongly on the route of Ca^2+^ entry into neurons. In particular, NMDAR-mediated Ca^2+^ influx appears to be especially efficiently coupled to mitochondrial Ca^2+^ loading compared with other Ca^2+^ entry pathways, as demonstrated in cultured hippocampal neurons (Stanika et al. 2009). These data indicate that MCU-dependent protection in CA2 is stimulus-dependent and appears to be preferentially associated with NMDAR-driven mitochondrial Ca^2+^ loading rather than with Glu exposure, in which stimulation is distributed across multiple glutamatergic receptor classes. Importantly, considering that in our experimental paradigm Glu exposure still induced robust CA1 injury, similarly to NMDA exposure, while sparing CA2 neurons, these findings additionally indicate that MCU is not the sole determinant of the unique resistance of CA2 neurons to excitotoxic injury. Further studies will be therefore required to identify additional molecular and cellular mechanisms contributing to the selective resistance of CA2 neurons under different excitotoxic conditions.

### Translational implications and future directions

Our findings may also have translational relevance for neurological disorders associated with excitotoxic injury, including stroke, epilepsy, and traumatic brain injury, in which mitochondrial Ca^2+^ dysregulation contributes to neuronal death (Zhang et al. 2019; Bierhansl et al. 2024; Liu et al. 2025). MCU has commonly been considered a potential therapeutic target in neurological disorders aimed at limiting mitochondrial Ca^2+^ overload (Rodríguez et al. 2022; Johnson et al. 2023). However, our data indicate that the consequences of MCU inhibition may differ substantially between neuronal populations and types of excitotoxic stimuli. Given the substantial evidence that mitochondrial Ca^2+^ overload can be deleterious in many neuronal populations, any therapeutic modulation of MCU will likely need to be highly cell‑type‑, region‑ and context‑specific, and our findings in CA2 should not be interpreted as a general argument for MCU activation as a universal neuroprotective strategy.

Notably, while MCU inhibition sensitized CA2 neurons to NMDA-induced injury, it did not enhance CA2 vulnerability to Glu-induced excitotoxicity under conditions resulting in comparable CA1 damage. These findings suggest that the role of MCU in neuronal survival is highly variable depending on the cellular environment and pathological setting. They also highlight the importance of considering the regional specificity of Ca^2+^ handling and cellular MCU function when developing therapeutic strategies targeting MCU.

Although pharmacological MCU inhibition allowed us to identify a potential contribution of MCU to CA2 resistance, future studies employing complementary genetic approaches will be required to further validate the causal role of MCU in this phenomenon. Additional studies should also aim to establish an integrated picture of CA2-specific intra- and extra-mitochondrial Ca^2+^ homeostasis. Particular attention should be paid to the potential interaction between MCU and other CA2-enriched Ca^2+^-regulating mechanisms, including cytosolic Ca^2+^ buffering by Ca^2+^-binding proteins and enhanced Ca^2+^ extrusion systems. It will also be important to determine how NMDA-induced, MCU-mediated mitochondrial Ca^2+^ uptake influences mitochondrial function in CA2 neurons, including its effect on mitochondrial membrane potential, respiration and energy production. In addition, it remains to be determined whether CA2 pyramidal cells possess specific regulatory mechanisms controlling MCU-dependent and MCU-independent mitochondrial Ca^2+^ fluxes and how these mechanisms differ in comparison with CA1 neurons. Finally, comparative studies will be required to explore differential involvement of MCU in protection against excitotoxicity mediated by different classes of glutamatergic receptors in CA2.

## Conclusions

Our findings identify a strong association between MCU activity and the selective resistance of CA2 neurons to NMDA-induced excitotoxicity. We show that MCU levels are inversely correlated with excitotoxic sensitivity across hippocampal CA subregions, with CA2 showing the highest MCU expression and lowest vulnerability. MCU levels in CA2 further increases after NMDA exposure, suggesting stress-induced upregulation and possible involvement in CA2 neuron protection. Supporting this, pharmacological MCU inhibition sensitizes CA2 neurons to NMDA in a dose-dependent manner, demonstrating a functional link between MCU activity and neuroprotection against NMDA excitotoxicity in this region. In contrast, MCU blocking did not sensitize CA2 neurons to Glu-induced excitotoxicity under conditions producing comparable CA1 damage, indicating that the contribution of MCU to neuronal survival may depend on the type of excitotoxic stimulus. These results support the hypothesis that MCU acts as a CA2‑enriched factor associated with the long-known resistance of CA2 neurons to excitotoxicity (Dudek et al. 2016), particularly during NMDA-mediated injury, while the exact mitochondrial mechanisms remain to be elucidated. These findings also open interesting directions for future studies aimed at understanding the mechanistic role of MCU in CA2 neuroprotection using genetic approaches and direct assessment of mitochondrial function. Considering the translational implications, our data further suggest that therapeutic strategies targeting MCU and mitochondrial Ca^2+^ handling in neurological disorders should carefully consider the diverse and potentially opposing neuronal responses to MCU modulation, depending both on neuronal population and the type of excitotoxic stimulus.

## Data Availability

The datasets generated and analysed during the current study are available in the RepOD repository, https://repod.icm.edu.pl/dataset.xhtml? persistentId=doi:10.18150/P9YKB2.

## Abbreviations

AMPA: α-amino-3-hydroxy-5-methyl-4-isoxazolepropionic acid
ANOVA: Analysis of Variance
DAB: Diaminobenzidine
DIV: Day In Vitro
Glu: Glutamate
LTD: Long-term Depression
LTP: Long-term Potentiation
MCU: Mitochondrial calcium uniporter
NMDA: N-methyl-D-aspartate
NMDAR: N-methyl-D-aspartate receptor
PI: Propidium Iodide
RGS: 14-Regulator of G-protein signaling 14
